# Data on microbial and physiochemical characteristics of inlet and outlet water from household water treatment devices in Rasht, Iran

**DOI:** 10.1016/j.dib.2017.12.038

**Published:** 2017-12-20

**Authors:** Dariush Naghipour, Seyed Davoud Ashrafi, Ali Mojtahedi, Masoud Vatandoost, Loghman Hosseinzadeh, Esmail Roohbakhsh

**Affiliations:** a School of Health, Guilan University of Medical Sciences, Rasht, Iran; b Research Center of Health and Environment, Guilan University of Medical Sciences, Rasht, Iran; c Department of Microbiology, Faculty of Medicine, Guilan University of Medical Sciences, Rasht, Iran; d Department of Biology, Science and Research Branch, Islamic Azad University, Tehran, Iran

**Keywords:** Household water treatment, Drinking water quality, Anions, Cations

## Abstract

In this research, we measured various parameters related to drinking water quality include turbidity, temperature, pH, EC, TDS, Alkalinity, fecal and total coliform, heterotrophic plate count (HPC), free chlorine, Mn, Ca, Mg, Fe, Na, Cl^−^, F^−^, HCO_3_, in the inlet and outlet of household water treatment devices according to the standard methods for the examination of water and wastewater (W.E. Federation and Association and A.P.H., 2005) [1]. Sixty four inlet and outlet water samples were taken from thirty two household water treatment devices from eight different residential blocks in Golsar town of Rasht, Iran. The data obtained from experiments were analyzed using the software Special Package for Social Sciences (SPSS 24) and MS-Excel.

**Specifications table**

**Table:** 

Subject area	Environmental Engineering
More specific subject area	Drinking water quality
Type of data	Figure and table
How data was acquired	Total dissolved solid (TDS) were measured by scaling method using oven and digital scale.
	Anions and Cations were measured by using UV–vis spectrophotometer and flame photometer.
	Total and fecal coliform were determined by multiple-tube fermentation technique.
	Heterotrophic plate count (HPC) was done using membrane filtration method.
	Free chlorine was measured using DPD method.
Data format	Raw, analyzed.
Experimental factors	Samples were collected randomly from eight blocks in Golsar town of Rasht. The glasses bottles (250 ml and 2000 ml) were used to samples collection. The samples were taken transferred to the laboratory under acidic condition and 4 °C for analyzing of anions and cations. Although, for analyzing of microbial parameters the samples were transferred under 6 h and the temperature of 4 °C.
Experimental features	Physicochemical and microbial parameters of drinking water include; K^+^, $NO_{3}^{-}$, Mn^2+^, Mg^2+^, Ca^2+^, Na^+^, Cl ^−^, Fe^2+^, Mg^2+^, F^−^, HCO_3_, TDS, Ec, pH, turbidity, total hardness, alkalinity, free chlorine, temperature, total and fecal coliform and HPC.
Data source location	Golsar town of Rasht, Guilan Province, Iran.
Data accessibility	All data are available within this article.

**Value of the data**

• These data describe performance of household water treatment device and will be useful for who use this devices for water purification.
• The data will be valuable for the experts of healthcare center.
• The data will be useful for the engineers related to household water treatment device maintenance.

## 1 Data

The data in this paper express the quality of urban drinking water and household water in the inlet and outlet of household water treatment devices. So, the selected parameters of drinking water quality were some important microbial and physiochemical parameters such as; K^+^, $NO_{3}^{-}$, Mn^2+^, Mg^2+^, Ca^2+^, Na^+^, Cl^−^, Fe^2+^, F^−^, HCO_3_, total and fecal coliform, turbidity, temperature, total hardness, TDS, EC, alkalinity, free chlorine and Heterotrophic plate count (HPC) [2], [3], [4], [5], [6]. The data from the experiments of inlet water for physicochemical parameters; turbidity, temperature, EC, pH, total hardness and total alkalinity were 0.73 NTU, 23.1 °C, 587 µs/cm, 7.62, 182.5 mg/L CaCO_3_ and 190.1 mg/L CaCO_3_, respectively (Table 1). Although, the value of these parameters in outlet were 0.26 NTU, 23.9 °C, 124 µs/cm, 6.95, 56.4 mg/L CaCO_3_ and 53.7 mg/L CaCO_3_, respectively (Table 1). Aimed at the microbial quality of inlet water the data from the experiments for parameters; fecal and total coliform, heterotrophic plate count (HPC) and free chlorine were 0 and 0.4 MPN/100 mL, 7 CFU/mL and 0.2 mg/L, respectively (Table 2). While, the value of these parameters in outlet water were 0.2 and 0.9 MPN/100 mL, 324 CFU/mL and 0 mg/L, respectively (Table 2). In addition, values of inlet cations and anions parameters; Mn^2+^, Ca^+2^, Mg^2+^, Na^+^, K^+^, Fe^2+^, $NO_{3}^{-}$, Cl^−^, F^−^ and HCO_3_ were 0.07, 47.9, 14.1, 31.7, 0.51, 0.13, 1.1, 63.7, 0.03 and 230.8 mg/L, respectively (Table 3). Although, values of outlet were 0.0025, 14.1, 6.9, 11.2, 0.1, 0.05, 0.69, 22.7, 0.02 and 65.5 mg/L, respectively (Table 3). According to data, the microbial quality of outlet water of household water treatment device was decreased and could not provide WHO standards (Table 2). Statistical analysis of data for inlet and outlet water quality were presented in Table 4, Table 5.

**Table 1:** The values of physicochemical parameters in inlet and outlet of household water treatment device.

**Parameter**	**Unit**	**Mean**		**Standard deviation**		**Removal efficiency (%)**	**Standards**		
		**Inlet**	**Outlet**	**Inlet**	**Outlet**		**Iran standard**	**EPA**	**WHO**
Turbidity	NTU	0.73	0.26	0.2	0.08	10.2	5	5	5
Temperature	C	23.1	23.9	1.5	1.5	–	–	–	–
pH	–	7.62	6.95	0.14	0.28	–	6.5–9	6.5–8.5	6.5–8.5
EC	µs/cm	587	124	125	82.7	79	–	–	–
Total hardness	mg/l CaCo_3_	182.5	56.4	11.3	23.8	69	500	–	500
Total alkalinity	mg/l CaCo_3_	190.1	53.7	48.3	36.8	71.6	–	–	–

**Table 2:** The values of microbial parameters in inlet and outlet of household water treatment device.

**Parameter**	**Unit**	**Mean**		**Standard deviation**		**Removal efficiency (%)**	**Standards**		
		**Inlet**	**Outlet**	**Inlet**	**Outlet**		**Iran standard**	**EPA**	**WHO**
Total coliform	MPN/100	0.4	0.9	0.56	0.64	–	0	0	0
Fecal coliform	MPN/100	0	0.2	0	0.6	–	0	0	0
HPC	CFU/mL	7	324	4.17	134	–	<500	<500	<500
Free chlorine	mg/L	0.2	0	0.12	0	–	5	4	5

**Table 3:** The values of cations and anions parameters in inlet and outlet of household water treatment device.

**Parameter**	**Unit**	**Mean**		**Standard deviation**		**Removal efficiency (%)**	**Standards**		
		**Inlet**	**Outlet**	**Inlet**	**Outlet**		**Iran standard**	**EPA**	**WHO**
Manganese	mg/L	0.07	0.0025	0.06	0.007	91.7	0.05	0.05	0.4
Calcium	mg/L	47.9	14.1	7.1	3.9	70.6	200	–	200
Magnesium	mg/L	14.1	6.9	3.9	5.8	65.2	150	–	–
Sodium	mg/L	31.7	11.2	10	9.3	64.6	200	200	200
Potassium	mg/L	0.51	0.1	0.56	0.19	80.3	–	–	–
Iron	mg/L	0.13	0.05	0.02	0.06	65.4	0.3	0.3	0.3
Nitrate	mg/L	1.1	0.69	1.14	1.36	37	45($NO_{3}^{-}$)	10(N)	10(N)
Chloride	mg/L	63.7	22.7	16.2	7.7	64.4	400	250	250
Fluoride	mg/L	0.03	0.02	0.04	0.03	44.4	1.5	2	1.5
bicarbonate	mg/L	230.8	65.5	57.7	44.9	71.6	–	–	–

**Table 4:** Paired statistical analysis for comparing inlet and outlet data.

**Parameter**	**Unit**	**t-test**	**Degrees of freedom**	***P*-value**
HPC	CFU/mL	−6.312	7	0
Turbidity	Mg/L	6.023	7	0.001
TDS	Mg/L	8.774	7	0
Total alkalinity	Mg/L	7.676	7	0
Total Hardness	Mg/L	12.766	7	0
Calcium	Mg/L	10.780	7	0
Iron	Mg/L	5.534	7	0
Chloride	Mg/L	8.116	7	0.001
Sodium	Mg/L	9.988	7	0
EC	Mg/L	8.775	7	0

**Table 5:** Paired statistical analysis results for comparing inlet and outlet data.

**Parameter**	**Unit**	**z-test**	**Mann–Whitney Test**	***P*-value**
Total coliform	MPN/100 mL	−1.541	18	0.123
Fecal coliform	MPN/100 mL	−1	28	0.317
Free chlorine	Mg/L	−2.919	8	0.004
Magnesium	Mg/L	−2.731	6	0.006
Manganese	Mg/L	−2.176	14	0.03
Nitrate	Mg/L	−1.042	23	0.298
Fluoride	Mg/L	−0.544	8	0.586
Potassium	Mg/L	−2.551	8	0.011
pH	–	−2.524	8	0.012

## 2 Experimental design, materials and method

### 2.1 Study area description

**Fig. 1:**
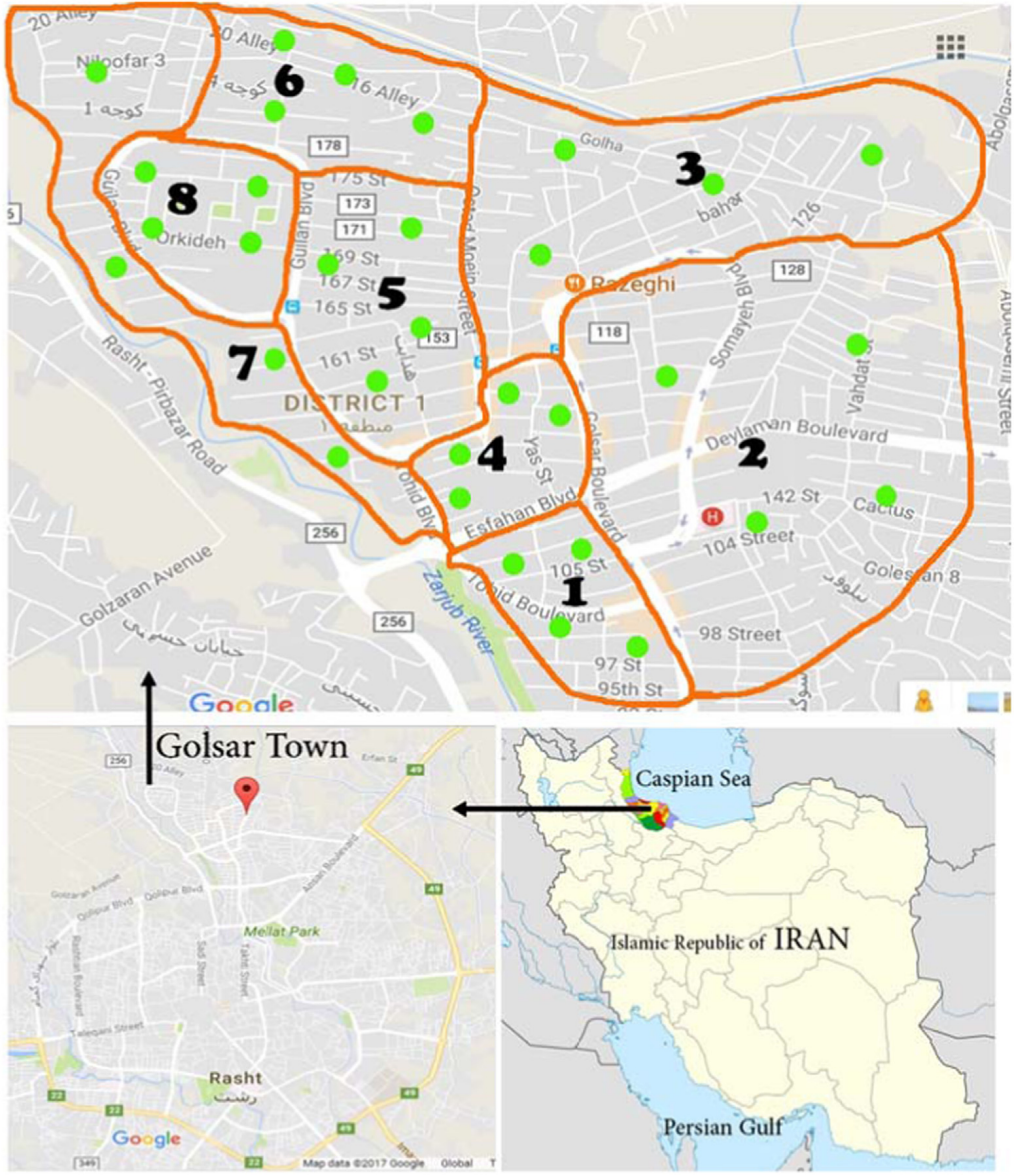
Map and sampling points of study area.

According to map and sampling points on Fig. 1, the study site is localized to eight blocks in Golsar town of Rasht in Guilan Province, Iran. In each block four devices in four different homes has been selected. All household water treatment devices contains; cotton fibers, ion exchange cartridge, carbon active cartridge, and reverse osmosis.

### 2.2 Sample collection and analytical procedure

Sampling and experimental period were in April to June which collected and analyzed according to the standard method [1]. The data were analyzed using the software Special Package for Social Sciences (SPSS 24) and MS-Excel.

## References

[1] W. E. Federation and Association, A.P.H. (2005). Standard Methods for the Examination of Water and Wastewater.

[2] Yousefi N., Fatehizedeh A., Ghadiri K., Mirzaei N., Ashrafi S.D., Mahvi A.H. (2016). Application of nanofilter in removal of phosphate, fluoride and nitrite from groundwater. Desalin. Water Treat..

[3] Soltani F., Ghomeishi A., Mohammadi M.J., Karimyan A., Khoshgoftar M., Darabpour F., Afkar A., Yari A.R., Mahboubi M., Rastegarimehr B., Yusefzadeh A., Salehi S.Z., Vosoughi M., Geravandi S. (2017). Association of toxic microbial and chemical water quality of hemodialysis instruments during 2016. Fresenius Environ. Bull..

[4] Ashrafi S.D., Kamani H., Mahvi A.H. (2016). The optimization study of direct red 81 and methylene blue adsorption on NaOH-modified rice husk. Desalin. Water Treat..

[5] Ashrafi S.D., Kamani H., Soheil Arezomand H., Yousefi N., Mahvi A.H. (2016). Optimization and modeling of process variables for adsorption of Basic Blue 41 on NaOH-modified rice husk using response surface methodology. Desalin. Water Treat..

[6] Ashrafi S.D., Kamani H., Jaafari J., Mahvi A.H. (2016). Experimental design and response surface modeling for optimization of fluoroquinolone removal from aqueous solution by NaOH-modified rice husk. Desalin. Water Treat..

